# Atomic-Scale Layer-by-Layer Deposition of FeSiAl@ZnO@Al_2_O_3_ Hybrid with Threshold Anti-Corrosion and Ultra-High Microwave Absorption Properties in Low-Frequency Bands

**DOI:** 10.1007/s40820-021-00678-4

**Published:** 2021-07-30

**Authors:** Wei Tian, Jinyao Li, Yifan Liu, Rashad Ali, Yang Guo, Longjiang Deng, Nasir Mahmood, Xian Jian

**Affiliations:** 1 National Engineering Researching Centre of Electromagnetic Radiation Control Materials, Key Laboratory of Multi-Spectral Absorbing Materials and Structures of Ministry of Education, State Key Laboratory of Electronic Thin Films and Integrated Devices, School of Electronic Science and Engineering, University of Electronic Science and Technology of China, Chengdu, 611731 People’s Republic of China; 2 School of Electronic Science and Engineering, The Yangtze Delta Region Institute (Huzhou, University of Electronic Science and Technology of China, Huzhou, 313001 People’s Republic of China; 3 School of Materials and Energy, University of Electronic Science and Technology of China, Chengdu, 611731 People’s Republic of China; 4 School of Electrical and Information Engineering, Panzhihua University, Panzhihua, 617000 People’s Republic of China; 5 School of Engineering, RMIT University, Melbourne, Victoria 3001 Australia

**Keywords:** Atomic layer deposition, Magnetic alloy, Dual-oxide-shells, Microwave absorption, Anti-corrosion

## Abstract

Developing highly efficient magnetic microwave absorbers (MAs) is crucial, and yet challenging for anti-corrosion properties in extremely humid and salt-induced foggy environments. Herein, a dual-oxide shell of ZnO/Al_2_O_3_ as a robust barrier to FeSiAl core is introduced to mitigate corrosion resistance. The FeSiAl@ZnO@Al_2_O_3_ layer by layer hybrid structure is realized with atomic-scale precision through the atomic layer deposition technique. Owing to the unique hybrid structure, the FeSiAl@ZnO@Al_2_O_3_ exhibits record-high microwave absorbing performance in low-frequency bands covering L and S bands with a minimum reflection loss (RL_min_) of -50.6 dB at 3.4 GHz. Compared with pure FeSiAl (RL_min_ of -13.5 dB, a bandwidth of 0.5 GHz), the RL_min_ value and effective bandwidth of this designed novel absorber increased up to ~ 3.7 and ~ 3 times, respectively. Furthermore, the inert ceramic dual-shells have improved 9.0 times the anti-corrosion property of FeSiAl core by multistage barriers towards corrosive medium and obstruction of the electric circuit. This is attributed to the large charge transfer resistance, increased impedance modulus |Z|_0.01 Hz_, and frequency time constant of FeSiAl@ZnO@Al_2_O_3_. The research demonstrates a promising platform toward the design of next-generation MAs with improved anti-corrosion properties.

**Supplementary Information:**

The online version contains supplementary material available at 10.1007/s40820-021-00678-4.

## Highlights

- A multiscale structure is realized through layer-by-layer deposition with atom-scale precision via atomic layer deposition
- FeSiAl@ZnO@Al_2_O_3_ exhibits record-high absorption properties in low-frequency bands.
- The corrosion resistance is improved by the unique multistage oxide barriers.

**Supplementary Information:**

The online version contains supplementary material available at 10.1007/s40820-021-00678-4.

## Graphical abstract

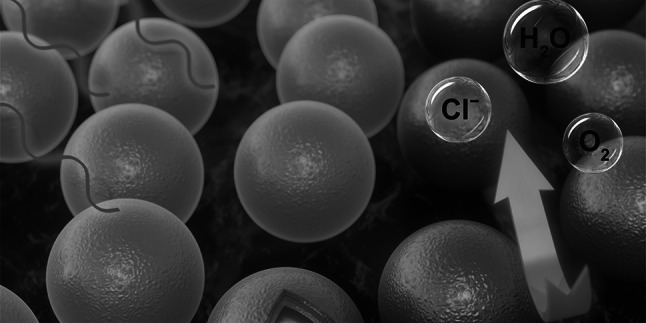

## Introduction

Since the investigation into microwave absorbers (MAs) in the 1940s, developing highly effective MAs with “thin, light, wide and strong” characteristics has been a major challenge so far to deal with electromagnetic pollution and realizing strength for military and civil purposes [1–6]. Generally, the impedance matching and attenuation characteristics of MAs are key for effective microwave absorption properties [7]. Among various designed MAs, FeSiAl (FSA) alloy has gained extensive attention due to low cost, high saturation magnetization, superior magnetic permeability, and strong absorption characteristics [8, 9]. However, FSA suffers from the ease of corrosion, magnetic aggregation and possess high density, which hampers its practical applications [10, 11].

Furthermore, microwave absorption bandwidth and efficiency are limited by Rozanov (The minimum thickness of absorbers should not less than 1/17 of the wavelength corresponding to the minimum operating frequency) and Snoek’s limit (the resonant frequency of absorbers is inversely proportional to the static permeability), therefore, single ferrite magnetic MAs are difficult to possess excellent microwave absorption performance because of their poor impedance matching [12–14]. Until now, two typical strategies have been adapted to synthesize novel MAs; One is the designing of magnetic composites (dielectric/magnetic) with tunable electromagnetic parameters including pronounced impedance matching and excellent dielectric-magnetic loss properties. For example, Wang et al. developed a CoFe_2_O_4_/rGO@PVP composite with a minimum reflection loss (RL_min_) value of -56.8 dB with a matching thickness of 1.96 mm and an effective absorption bandwidth (EAB, RL < -10 dB) of 6.8 GHz [15]. Similarly, the microwave absorption property of CoNi alloy can be improved by making their composites through constructing unique core–shell and yolk-shell structures (like CoNi core, SiO_2_, and TiO_2_ shells), which achieved the RLmin value of -58.2 dB at 10.4 GHz [16]. Moreover, by introducing both dielectric ZnO and Al_2_O_3_ functional oxides better MAs can be developed due to their dipole relaxation and dielectric behaviors [17–19]. For instance, the conductivity of ZnO film is enhanced when doped with Al_2_O_3_ in effect with impedance matching [20]. Where investigations have confirmed that constructing hollow Ni-Al_2_O_3_-ZnO through atomic layer deposition (ALD) gives RL_min_ of –50 dB at 9.44 GHz, much better than that of Ni-Al_2_O_3_ nanowires [21]. In the same way, Liu et al., harvest proper EM matching and multi-polarization by developing Fe@ZnO composites that showed RL_min_ of -57.1 dB at 7.8 GHz [22]. The other strategy is preparing MAs with specific morphology or microstructure such as flower-like 3D Co_3_O_4_-rGO hybrid-architectures, Fe_3_O_4_@C nanorings, Co_1–x_S hollow spheres, and 3D Fe/C porous nanofibers for improving microwave absorption performances due to their dielectric and crystalline anisotropies [23–26]. This research progress indicated that suitable impedance matching, high saturation magnetization, and crystalline anisotropy have a significant impact on microwave absorbing properties, especially, in the low-frequency range of L and S bands (1.0–4.0 GHz). Therefore, to develop effective MAs for low-frequency range, these characteristics should be controlled through engineering the structure, composition and right match of different materials in hybrid nanostructures.

Nevertheless, traditional magnetic MAs (Fe, Co, Ni, and their alloys) have limitations in the complex corrosive and oxidative environments [10, 11]. Therefore, introducing anti-corrosion property is needed to investigate for the next-generation MAs. At present, some typical strategies are used to prevent corrosion such as active corrosion inhibition, anodic passivation, cathodic protection, and self-healing [27]. For example, Parsons et al. deposited ultrathin Al_2_O_3_, ZnO, TiO_2_, ZrO_2_, and HfO_2_ films on the Cu surface and verified the anti-corrosion properties in 0.1 M NaCl electrolyte. The results confirmed that ceramic oxides can protect the copper metal from Cl- ions and O_2_, and H_2_O molecules [28]. In the same way, Sahoo et al. reported that carbonyl iron microspheres coated with graphene oxide sheets (GO) using the ALD method possessed excellent corrosion resistance behavior [29]. They revealed that the passivation layer of GO sheets on the metal surface is highly efficient for protecting metal from erosion. In particular, ALD is a precise technique for the decoration of the passivation layer with preferable advantages including controlled interface, remarkable uniformity, and efficient thickness control. Currently, a large number of materials are being prepared through the ALD process such as dielectric absorbers (ZnO, Al_2_O_3_, ZrO_2_), and magnetic absorbers (Ni, Fe_3_O_4_, NiFe_2_O_4_) [30–33]. It is considered an accurate technology to design dielectric/magnetic composites with outstanding microwave absorption performances by adjusting the permittivity and permeability. However, regardless of extensive research is being conducted on microwave absorption, the bifunctional intrinsic connection between microwave absorption and corrosion protection has not been investigated yet.

In this work, a dual-oxide shell of ZnO/Al_2_O_3_ anchored on FeSiAl surface was designed in a layer-by-layer fashion with atomic-scale precision through the ALD process to demonstrate a useful strategy for the fabrication of highly efficient MAs with anti-corrosion properties. The results showed that the as-obtained absorbers exhibited dielectric-magnetic hybrid structure, well-matched impedance, and enhanced microwave attenuation ability with record-high absorption properties in low frequencies covering L and S bands. Especially, the FSA@ZnO@Al_2_O_3_ gradient structure achieves a high RL_min_ value of -50.6 dB at 3.4 GHz, covering the relatively low-frequency bands. The RL_min_ and EAB value of the novel absorber increased up to ~ 3.7 and ~ 3 times, respectively, than bare FSA alloy. Moreover, the ZnO/Al_2_O_3_ dual-oxide-shell also possesses 9 times better corrosion protection than that of a single ZnO or Al_2_O_3_ single-oxide-shell in 5.0 wt% NaCl solution. This technique provides a promising way for the designing of next-generation MAs with improved anti-corrosion properties.

## Experimental Section

### Materials

FeSiAl alloy (FSA) with particle size 2–4 µm shown in Fig. S1b was purchased from Changsha Tianjiu metal materials company (AR 99.99%).

### Synthesis of Dual-oxide-shell Encapsulated FeSiAl

Dual-oxide-shell encapsulated FSA was prepared by the ALD technique. FSA particles were dispersed in ethanol by sonicating for 1 min, then dispersed onto an alumina substrate and air dried. Afterwards, the ZnO layer was deposited on FSA at 150 ℃ with N_2_ as a carrier gas, using diethyl zinc (DEZn, Zn(C_2_H_5_)_2_) and deionized H_2_O as the Zn and O source, respectively. DEZn and H_2_O were introduced into the reaction chamber with the sequence of ABAB about 250 cycles, the dose and purging time were 0.02 and 8 s, respectively. Subsequently, the Al_2_O_3_ shell was deposited using trimethylaluminum (TMA, Al (CH_3_)_3_) as an Al source, following the same steps and conditions as for ZnO deposition. Finally, the collected sample was transferred to a furnace and annealed at 500 ℃ under an N_2_ atmosphere for about 2 h. The sample was named FSA@ZnO@Al_2_O_3_. The single ZnO and single Al_2_O_3_ shells were deposited on the FeSiAl at 150 °C with the similar conditions of FSA@ZnO@Al_2_O_3_ as a comparison, named as FSA@ZnO and FSA@Al_2_O_3_, respectively. In addition, FSA alloy was transferred to a furnace and annealed at 500 ℃ under an N_2_ atmosphere for about 2 h as a control sample, named FSA-500.

### Characterization

X-ray diffraction (XRD) was used to identify the crystal structure of FSA and FSA-based absorbers. The morphology, microstructure, and elements distribution were measured using field-emission scanning electron microscopy (FESEM, JSM-7600F) and high-resolution transmission electron microscopy (HRTEM, FEI Tecnai). The surface compositions and element valence state of FSA@Al_2_O_3_@ZnO gradient structure were scanned by X-ray photoelectron spectroscopy (XPS). Besides, the magnetic properties of FSA and as-prepared materials were characterized using the physical property measurement system (PPMS) at room temperature. The related electromagnetic parameters were measured by an N5234A vector network analyzer in 0.5–18 GHz. The electrochemical behaviors of those samples were characterized using an electrochemical workstation in 5.0 wt% NaCl solution with a typical three-electrode system (working electrode: FSA and FSA-based absorbers, counter electrode: Pt foil and reference electrode: Ag/AgCl). The testing working electrodes were fabricated by filling the absorbers into polyvinylidene difluoride (PVDF) binder with a mass ratio of 4:1 in the form of plate-like samples. The open-circuit potential (OCP) was measured for ~ 1600 s to achieve a balanced state of the electrochemical systems, then the Tafel test and electrochemical impedance spectroscopy (EIS) were tested. The Tafel test was conducted in -500–500 mV vs. OCP with a scan rate of 1 mV s^−1^. The EIS measurements were carried out at OCP over 10^5^–10^−2^ Hz with 5 mV perturbation potential. Also, the recorded data were fitted through the ZSimpWin software to achieve corresponding corrosion parameters.

## Results and Discussion

### Fabrication and Characterization of FSA-based Absorbers

**Fig. 1:**
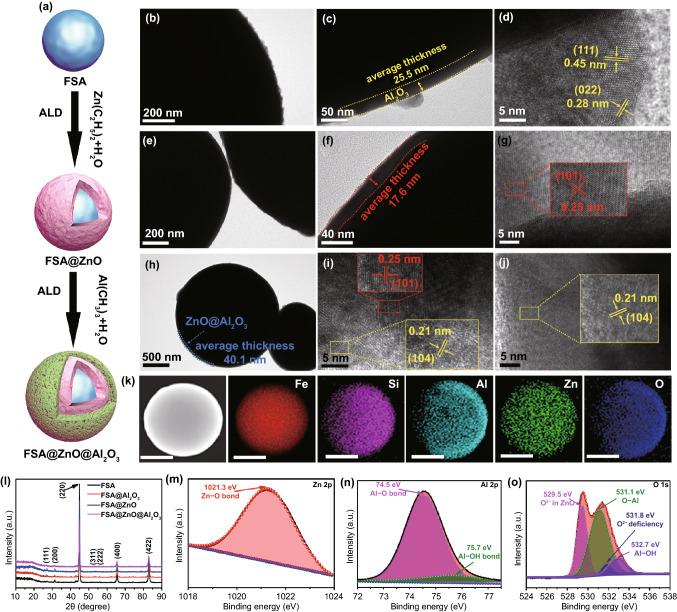
**a** Schematic illustration of the FSA@ZnO@Al_2_O_3_ gradient structure. HRTEM images of **b**–**d** FSA@Al_2_O_3_ core–shell structure, **e**–**g** FSA@ZnO core–shell structure, **h**–**j** FSA@ZnO@Al_2_O_3_ gradient structure. **k** TEM element distribution of FSA@ZnO@Al_2_O_3_ gradient structure. The scale bar in all Figures is 1 μm. **l** XRD patterns. High-resolution XPS spectra of FSA@ZnO@Al_2_O_3_ gradient structure: **m** Zn 2p, **n** Al 2p, **o** O 1s elements

The FSA@ZnO@Al_2_O_3_ core–shell hybrid is synthesized using ALD approach as described schematically in Fig. 1a. Before putting dual protection, individual coatings were carried out to observe their growth and compatibility with FSA. The morphology of FSA@Al_2_O_3_ core–shell structure is displayed in Fig. 1b-d, showing a uniform Al_2_O_3_ shell with an average thickness of ~ 25.5 nm over the FSA core. The lattice spacings of 0.28 and 0.45 nm were observed in HREM analysis corresponds to (022) and (111) planes of Al_2_O_3_ shell, respectively [34]. Similarly, ZnO shell deposition on FSA core results in a smooth surface with an average thickness of ~ 17.6 nm, showing a lattice spacing of 0.25 nm, assigned to the (101) plane of the ZnO as shown in Fig. 1e-g [35]. Finally, the dual coating was carried out to obtain FSA@ZnO@Al_2_O_3_ gradient structure having an average thickness of ~ 40.1 nm for ZnO/Al_2_O_3_ dual-oxide-shell as shown in Fig. 1h. The lattice spacing of 0.25 nm is observed at the inner side of the shell assigned to the (101) plane of ZnO, while the outer side showed an interlayer spacing of 0.21 nm assigned to (104) plane of Al_2_O_3_ as shown in Fig. 1i-j [36]. Interestingly, no interface mismatch between ZnO and Al_2_O_3_ shells was observed, assuring the construction of a very fine heterojunction. The elemental maps of FSA@ZnO@Al_2_O_3_ are displayed in Figs. 1k and S2, Fe, Si, Al, Zn, and O elements were evenly distributed around the microsphere. The concentrations of different elements for FSA@ZnO@Al_2_O_3_ in Fig. S3 and Table S1 revealed the low content of ZnO and Al_2_O_3_. Overall, the FSA@ZnO@Al_2_O_3_ gradient structure is constituted of inner FSA core, middle ZnO shell, and outer Al_2_O_3_ shell, forming a distinctive coaxial multi-interface layer-by-layer structure.

The XRD results show that the pure FSA consisted of a typical body-center cubic (bcc) structure, with peaks located at (31.4°), (44.9°), (53.3°), (55.9°), (65.5°), and (83.1°) are corresponding to bcc B2 phase (200), (220), (311), (222), (400), and (422) planes, respectively, according to the JCPDS No. 45–1206, presented in Figs. 1 and S4a. Interestingly, after annealing at 500 ℃, the (111) plane appeared in FSA-based hybrids, suggesting the formation of the DO_3_ superlattice structure [37]. However, the diffraction peaks of Al_2_O_3_ and ZnO are not found, which might be due to their lower content and beyond the detection limit of XRD. Therefore, XPS measurements were used to identify the existence of ZnO and Al_2_O_3_ in FSA@Al_2_O_3_@ZnO hybrid structure. The high-resolution Zn 2p showed a single Zn 2p_3/2_ peak at 1021.3 eV, corresponding to the Zn–O bond of the stoichiometric ZnO, evident in the formation of ZnO on the surface of FSA alloy (Fig. 1m). Similarly, Al 2p spectra could be divided into two peaks, the strongest peak locating at 74.5 eV originates from Al-O bonds, the other peak at 75.7 eV is associated with Al–OH hydroxyl groups [38, 39]. Besides, the high-resolution O 1s spectrum shows four chemical states of oxygen positioned at 529.5, 531.1, 531.8, and 532.7 eV, attributed to O^2−^ ions in ZnO matrix, O–Al bonds, oxygen-deficient areas in ZnO matrix, and Al–OH hydroxyl groups, respectively, as shown in Fig. 1o [35, 38–41]. So, the results of XRD and XPS indicate that FSA@Al_2_O_3_, FSA@ZnO, and FSA@ZnO@Al_2_O_3_ with DO_3_ superlattice structure were successfully synthesized through the ALD approach and annealing treatment.

Further, the M-H curves show an S-like shape with apparent coercivity and remanence, which proved well ferromagnetic behavior of hybrid (Fig. S4b). The Ms values of FSA, FSA-500, FSA@Al_2_O_3_, FSA@ZnO, and FSA@ZnO@Al_2_O_3_ are 119.4, 134.4, 123.3, 123.5, and 124.4 emu/g, respectively. A slight increase of M_s_ for FSA-based hybrids might attribute to the highly ordered DO_3_ superlattice structure [37]. The H_c_ values obtained in FSA, FSA@Al_2_O_3_, FSA@ZnO, and FSA@ZnO@Al_2_O_3_ are about 21.6, 27.0, 7.15, and 22.9 Oe, respectively, which are larger than the bulk Fe (1 Oe) might be due to smaller size [42]. In addition, the difference of H_c_ values among different samples might be due to the decrease of shape anisotropy, appropriate particle/crystal size, and the enhancement of crystallinity of FeSiAl components induced by the thermal treatment process [43]. To evaluate the electromagnetic wave absorbing properties, the RL values are calculated using complex permittivity (ε_r_ = ε′- iε′′) and permeability (μ_r_ = μ′- iμ′′) in the frequency range of 0.5–18 GHz and layer thickness of 0.5–5.0 mm based on the transmission line theory [44].

### Microwave Absorption Ability and Anti-corrosion Properties of FSA-based Absorbers

**Fig. 2:**
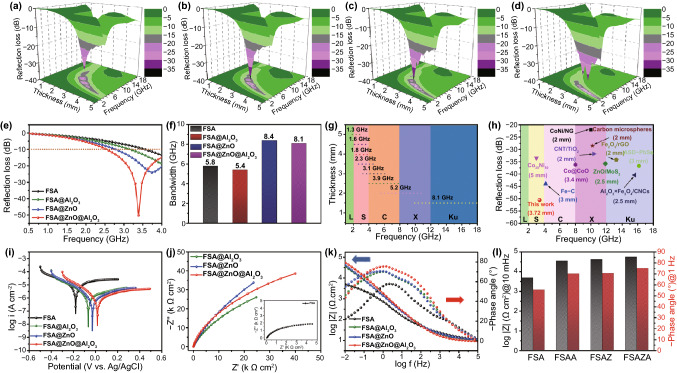
3D RL maps of as-prepared absorbers of d at 0.5–5.0 mm in 0.5–18 GHz: **a** FSA, **b** FSA@Al_2_O_3_, **c** FSA@ZnO, **d** FSA@ZnO@Al_2_O_3_. **e** RL curves of FSA-based samples at a thickness of 3.72 mm in 0.5–4.0 GHz, **f** the effective absorption bandwidth at a thickness of 1.5 mm, **g** effective absorption range of FSA@ZnO@Al_2_O_3_ at different layer thicknesses, **h** performance comparison of several microwave absorbers which have attracted large attention, including Co_20_Ni_80_ [45], Fe–C [46], Co@CoO [47], CNT/TiO_2_ [48], CoNi/NG [49], Carbon microspheres [50], Fe_2_O_3_/rGO [51], ZnO/MoS_2_ [52], Al_2_O_3_ × Fe_3_O_4_/CNCs [18], ASD-PbSs [53]. **i** potentiodynamic polarization curves, **j** Nyquist plots, **k** Bode plots, **l** impedance at 10 mHz and phase at 1.0 Hz of FSA, FSA@Al_2_O_3_ (FSAA), FSA@ZnO (FSAZ), and FSA@ZnO@Al_2_O_3_ (FSAZA) gradient structure in 5.0 wt% NaCl solution

The absorption peaks of FSA-based absorbers move to a lower frequency in the L and S bands compared to FSA, especially FSA@ZnO@Al_2_O_3_ gradient structure as depicted in three-dimensional (3D) RL maps (Fig. 2). When the thickness is 3.0 mm, the FSA achieved the RL_min_ value of -32.1 dB at 6.2 GHz, and the EAB is 4.3 GHz from 4.3 to 8.6 GHz, corresponding to C bands (Fig. 2a). While FSA@Al_2_O_3_ reached the RL_min_ of -38.2 dB at 5.5 GHz with EAB of 4.1 GHz from 3.8 to 7.9 GHz, related to C bands (Fig. 2b) and the RL_min_ value of FSA@ZnO is -37.5 dB at 4.7 GHz and EAB is 3.5 GHz from 3.4 to 6.9 GHz (Fig. 2c). In contrast, the FSA@ZnO@Al_2_O_3_ gradient structure delivered the RL_min_ value of -33.6 dB at 3.2 GHz with a matching thickness of 4.0 mm and the EAB located in a lower frequency range from 2.2 to 4.3 GHz (Fig. 2d). Generally, strong microwave absorption at low frequency (< 4.0 GHz, L, and S bands) has been a major challenge in electromagnetic wave absorption. Interestingly, the strong microwave absorption performance of FSA@ZnO@Al_2_O_3_ gradient structure is located in L and S bands, indicating its suitability as an absorber for solving this critical problem.

The detailed microwave absorption performance analysis was carried out to observe the behavior of different FSA-based absorbers at low frequency (f ˂ 4 GHz), displayed in Fig. 2e. It is found that the FSA@ZnO@Al_2_O_3_ gradient structure achieved the strongest microwave absorption capability at 0.5 ~ 4.0 GHz than that of other absorbers. For instance, the RL_min_ value of FSA, FSA@Al_2_O_3_, and FSA@ZnO were -13.5, -19.1, and -24.4 dB at ~ 4.0 GHz with an EAB of 0.5, 0.9, and 1.3 GHz, respectively. While, FSA@ZnO@Al_2_O_3_ gradient structure showed an RL_min_ of -50.6 dB at 3.4 GHz with EAB of 1.5 GHz from 2.5 to 4.0 GHz, which clearly shows that hybrid has outperformed the other absorbers. Besides, the EAB is also enhanced at 1.5 mm, like FSA, FSA@Al_2_O_3_, FSA@ZnO, and FSA@ZnO@Al_2_O_3_ showed EAB of 5.8, 5.4, 8.4, and 8.1 GHz, respectively, shown in Fig. 2f. Importantly, the effective absorption bandwidth of FSA@ZnO@Al_2_O_3_ covers 1.8–18.0 GHz (L-Ku band) by adjusting the thickness from 1.5–5.0 mm (Fig. 2g). Instead of comparing the hybrid with FSA, Fig. 2h outlines the microwave absorption performance comparison of several MAs which have attracted great attention. The optimal RL values of the reported absorbers mainly focus on C, X, and Ku bands, however, FSA@ZnO@Al_2_O_3_ achieves a far low RL_min_ value at the S-band, highlighting its wide range use, especially in low frequency.

The electrochemical measurements (potentiodynamic polarization curves, EIS, and Bode plots) are carried out to investigate the corrosion protection role of the ZnO or Al_2_O_3_ shells. The potentiodynamic polarization curves are tested, which directly provide the stability behavior of FSA-based absorbers exposed to the corrosive solution. Figure 2i presents the Tafel curves after reaching an equilibrium state in 5.0 wt% NaCl solution. The Tafel plots of metallic materials provide information about corrosion susceptibility (corrosion potential (*E*_corr_)) and corrosion dynamic kinetics (corrosion current (*I*_corr_)) [54]. The FSA, FSA@Al_2_O_3_, FSA@ZnO, and FSA@ZnO@Al_2_O_3_ showed *E*_corr_ values of -0.1783, -0.0628, -0.0263, and 0.0191 V, respectively. It can be found that ZnO, Al_2_O_3_, and ZnO/Al_2_O_3_ dual-oxide shells brought more positive potential than single FSA, suggesting that surface protection helps in preventing corrosion, however, the best results are only possible when both oxides are applied in conjunction. Further, the anodic current density of metal dissolution/corrosion rate of FSA@Al_2_O_3_, FSA@ZnO, and FSA@ZnO@Al_2_O_3_ is at least 1.0 orders of magnitude lower than that of pure FSA, indicating the FSA core is well protected by the oxide shells. Figure 2j-k are showing the Nyquist and Bode plots of the bare FSA and FSA-based absorbers immersed in 5.0 wt% NaCl solution about 1.0 h. The |Z|_0.01 Hz_ value is usually considered a semi-quantitative indicator to evaluate the corrosion protection performance of shells [54, 55]. The |Z|_0.01 Hz_ values of FSA@ZnO@Al_2_O_3_ are the highest (double the bare FSA) values among the four samples, indicating the good barrier capability of the dual-oxide shell. Furthermore, the time constant in Bode plots is also a significant parameter to analyze the anti-corrosion properties, which is related to different electrochemical processes. It is generally accepted that the high-frequency time constant is regarded as the charge transfer process at the interface between shell and electrolyte, the medium constant is considered to be the hydroxide layer/metal electrode interface, and the low-frequency time constant represents metal electrode/electrolyte interface [56, 57]. For protected FSA-based absorbers, there are two to three-time constants due to shell/electrolyte interfaces and metal/electrolyte interfaces, while only one time constant in pure FSA, shown in Fig. S9. Moreover, an obvious increase of the phase angle at 1.0 Hz is found in protected FSA-based absorbers compared with bare FSA, as shown in Fig. 2l. These results clearly support the enhanced corrosion resistance by ZnO/Al_2_O_3_ dual-oxide-shell in 5.0 wt% of NaCl solution.

### Microwave Absorption Mechanism of FSA-based Absorbers

**Fig. 3:**
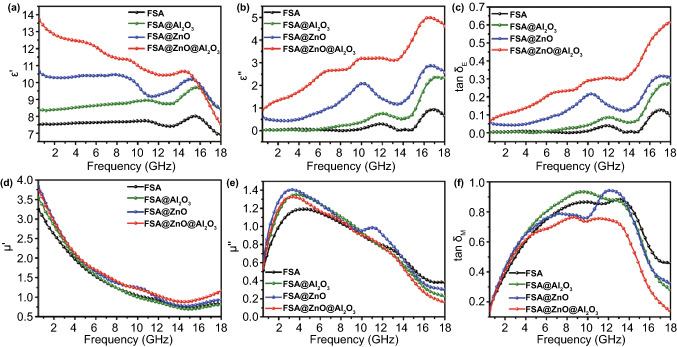
The frequency dependence of electromagnetic parameters of FSA alloy, FSA@Al_2_O_3_, FSA@ZnO, and FSA@ZnO@Al_2_O_3_: **a** real parts (ε′), **b** imaginary parts (ε′′), **c** dielectric loss tangents of the complex permittivity, **d** real parts (μ′), **e** imaginary parts (μ′′) and **f** magnetic loss tangents of the complex permeability

To understand the microwave absorption mechanism, electromagnetic parameters are important evidence to reflect the intrinsic properties of absorbers, including ε_r_ and μ_r_. As can be observed in Fig. 3a, after oxide shells decorated on FSA surface, the ε′ shows a rising trend in the whole frequency range of 0.5–18 GHz with the sequence of ε′ (FSA@ZnO@Al_2_O_3_) > ε′ (FSA@ZnO) > ε′ (FSA@Al_2_O_3_) > ε′ (FSA). Meanwhile, the ε′′ also demonstrates a similar trend with that of ε′-f curves shown in Fig. 3b, indicating the enhanced ability of storage and dissipation [21]. Furthermore, three resonance peaks appear at the frequency range of 6–8, 9–13, and 14–18 GHz in the ε′′-f curves for FSA@ZnO@Al_2_O_3_ gradient structure, which might result from interfacial polarization related to the existence of ZnO, Al_2_O_3_, and FSA alloy in the hybrid [21, 58]. Meanwhile, the existence of resonance peaks in the ε′′-f curves also indicates that the microwave dissipation capability of the gradient structure is unstable [53]. In addition, the relative complex permeability of FSA-500 is also enhanced, which confirmed the contribution of the thermal treatment process, as shown in Fig. S5. The improved complex permittivity might be attributed to the synergistic effects of the elimination of crystal defects and recrystallization of the FeSiAl alloy and interfacial polarization in the hybrid structure [59, 60]. The tanδ_E_ value (tanδ_E_ = ε′′/ε′) of FSA@ZnO@Al_2_O_3_ gradient structure is the highest among the four samples in 0.5–18 GHz, as shown in Fig. 3c, indicating dual-oxide-shells could enhance the dielectric loss capacity greatly. Figure 3d shows that the μ′ of as-prepared hybrids is slightly higher than that of FSA with 80 wt% filler loading. Meanwhile, the μ′′-f curves also exhibit a similar trend with the μ′-f curves in 0.5–18 GHz, especially in L, S, and C bands (Fig. 3e). Also, there are obvious peaks in 2.0–6.0 GHz, which are related to natural resonance [61]. Moreover, the μ′′ values of natural resonance peaks were enhanced clearly after annealing treatment. Generally, in the case of FSA alloy, there is a typical phase transformation from the A2 and B2 phase to DO_3_ phase under heat treatment. Thus, the well-ordered structure DO_3_ possesses higher saturation magnetization than that of A2 and B2 phases. These findings are well-supported by the XRD results, where the as-prepared FSA@Al_2_O_3_, FSA@ZnO, and FSA@ZnO@Al_2_O_3_ present an obvious diffraction peak of DO_3_ (111) compared to FSA, indicating the presence of a higher magnetic ordered phase of FSA-based hybrids. On the other hand, the highly ordered DO_3_ phase is contributed to acquiring stronger magnetic behavior [37]. Figure 3f shows magnetic loss tangents (tan δ_M_) of the complex permeability for FSA and FSA-based absorbers, the tan δ_M_ values of FSA@Al_2_O_3_ and FSA@ZnO are close to that of FSA. While the tan δ_M_ values of FSA@ZnO@Al_2_O_3_ gradient structure are smaller than that of FSA due to a certain amount of non-magnetic substance in the hybrid structure.

**Fig. 4:**
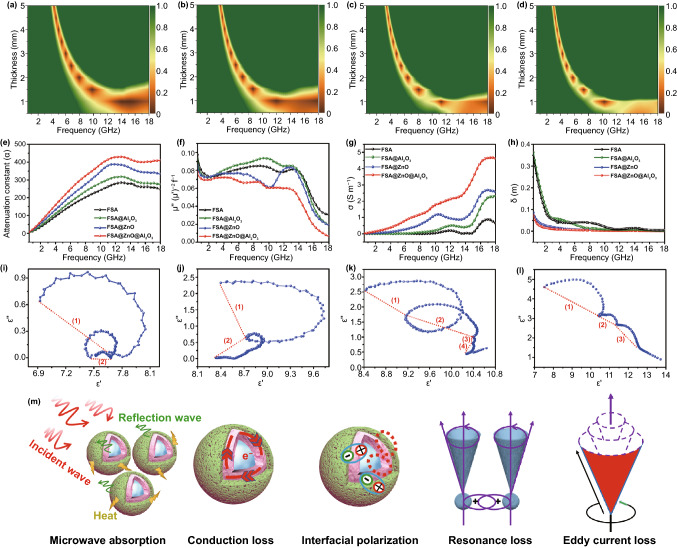
Calculated delta value maps of **a** FSA, **b** FSA@Al_2_O_3_, **c** FSA@ZnO, **d** FSA@ZnO@Al_2_O_3_. **e** Attenuation constants, **f** curves of μ"(μ')^−2^f^−1^ vs **f**, **g** electrical conductivity (σ), **h** skin depth (δ) of FSA and FSA-based samples. Typical ε′′- ε′ curves of four samples with 80 wt% filler content **i** FSA, **j** FSA@Al_2_O_3_, **k** FSA@ZnO, **l** FSA@ZnO@Al_2_O_3_. **m** The possible microwave absorption mechanism

It is generally accepted that two major factors are affecting microwave absorption i.e. impedance matching that allows more microwaves into the interior of absorbers when the impedance is equal or close to the free space and attenuation characteristics, including dielectric and magnetic loss. The description of A delta-function tool associated with the impedance matching characteristics is given in Note S1. Generally, a smaller delta value (|∆|< 0.4) means better impedance matching characteristics. As shown in Fig. 4a, the delta value (|∆|< 0.4) of bare FSA alloy covers the frequency range of 4.0–18 GHz with the thickness range of 0.5–5.0 mm, while the impedance matching is very poor in 0.5–4.0 GHz, which delineated the poor microwave absorbing properties in L and S bands. The FSA@Al_2_O_3_ also shows similar results with bare FSA, displayed in Fig. 4b. Interestingly, when ZnO is deposited on FSA, the impedance matching of FSA@ZnO and FSA@ZnO@Al_2_O_3_ are improved in L and S bands (Fig. 4c, d), which implies that more microwaves could easily enter into the absorbers to enhance microwave absorbing performance in L and S bands.

The attenuation characteristic is also a key factor to evaluate microwave absorption performance, making sure that the electromagnetic wave entering the absorber is rapidly attenuated. The equation of attenuation constant α is given in Note S1 and the results are presented in Fig. 4e. Bare FSA possesses the smallest α values among the four samples. While for dual-oxide-shell@FSA alloy, the attenuation constant α is enhanced in 0.5–18 GHz, which manifests that ZnO/Al_2_O_3_ dual-oxide-shell could improve the attenuation capability. To further study the attenuation mechanism of microwave absorption property, dielectric and magnetic loss are also investigated in detail. Generally, the magnetic loss mechanism is attributed to the eddy current effect, exchange resonance and natural resonance [19]. The eddy current loss can be explained by the equation C_0_ = μ"(μ')^−2^f^−1^≈2πμ_0_δd^2^/3, where δ is electric conductivity, d is the matching thickness [62]. Accordingly, if there is eddy current loss, the value of μ"(μ')^−2^f^−1^ vs frequency should be kept constant in a specific frequency range or the whole frequency range of 0.5–18 GHz. Figure 4f reveals the values of μ"(μ')^−2^f^−1^ for FSA@ZnO@Al_2_O_3_ keep constant in 6.6–8.8 GHz and 10.1–12.8 GHz, demonstrating that the eddy current loss mechanism makes a greater contribution in the gradient structure. Besides, the peaks located at 13.0 GHz are related to exchange resonance, which usually occurs at a high-frequency range [62]. Combined with the analysis of the μ" vs f curves, the peaks in 2.0–6.0 GHz might be due to natural resonance. Therefore, eddy current loss, exchange resonance and natural resonance are the main reasons for magnetic loss that affected the microwave absorption performance for the gradient structure in 0.5–18 GHz. Especially, natural resonance is beneficial with the enhanced performance of FSA@ZnO@Al_2_O_3_ gradient structure in low frequency covering L and S bands.

Dielectric loss property is another significant mechanism for microwave dissipation, which is usually investigated by Debye dipolar relaxation theory [63, 64]. Based on the deduction (Note S1), the deduction indicates that each semicircle represents a Debye relaxation process. Two cole–cole semicircles are found in Fig. 4i, j, indicating the effect of Debye relaxation loss on the improved dielectric properties of FSA/wax and FSA@Al_2_O_3_/wax composites. Moreover, after the deposition of ZnO and ZnO/Al_2_O_3_, as shown in Fig. 4k, l, there are more cole–cole semicircles than that of bare FSA, suggesting that more relaxations process exists in the FSA@ZnO/wax and FSA@ZnO@Al_2_O_3_/wax systems, such as electron polarization, interfacial polarization and Debye relaxation [53, 65]. The existence of interfaces in FSA-ZnO, FSA@ZnO-wax, ZnO-Al_2_O_3_, and FSA@ZnO@Al_2_O_3_-wax hybrids result in Maxwell–Wagner effect or interfacial polarization [50, 61, 66]. This phenomenon usually occurs in heterogeneous interfaces due to the accumulation of a high concentration of charges at the heterogeneous media and the production of many dipoles on FSA core or ZnO and Al_2_O_3_ shells. Similarly, electron polarization also contributes to the synergistic effect. Therefore, based on the free-electron theory $\varepsilon'' \, \propto \sigma /2\pi \varepsilon _{0} f$(σ, the electrical conductivity, $\sigma ~ = ~2\pi \varepsilon _{0} \varepsilon'' f$), an obvious increase in the values of the imaginary part for the complex permittivity indicated that the ZnO and Al_2_O_3_ shells with good crystal structure result in a large dielectric loss, as shown in Fig. 4g. Also, the skin depth (δ) of the electromagnetic wave irradiation shown in Fig. 4h is calculated with $\delta = \left( {\pi f\mu _{0} \mu _{r} \sigma } \right)^{{ - 1/2}}$, suggesting that a skin effect is clearly suppressed by ZnO shells in the low-frequency region, which is one of the reasons for the enhanced microwave absorption performance in L and S bands [67].

Besides dielectric and magnetic loss mechanisms, the quarter-wavelength matching model should not be ignored to explain the microwave absorption properties. According to the quarter-wavelength cancellation model, the RL_min_ value usually takes place when the thickness (d_m_) and the matching frequency (f_m_) obey the equation of $d = nc/4f\sqrt {\left| {\varepsilon _{r} } \right|\left| {\mu _{r} } \right|} ~(n = 1,3,5,~ \ldots )$ [42]. The reflected microwaves from the absorbers-metal interface and free space-absorbers interface are out of phase by 180° compared to incident microwaves, which could lead to a counterbalance of reflected waves on the interface of free space-absorbers. Figure S8 displays that the experimental d_m_ results are in good agreement with the simulated peak frequency using the quarter-wavelength (λ/4) cancellation model, demonstrating that the quarter-wavelength cancellation is also contributing to achieving excellent microwave absorption performance.

### Anti-Corrosion Mechanism of FSA-based Absorbers

**Fig. 5:**
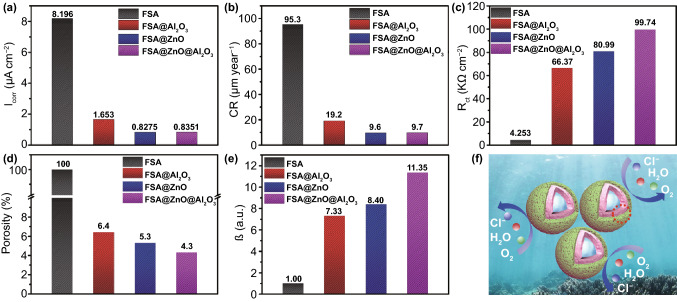
**a** corrosion current *I*_corr_, **b** corrosion rate, **c** transfer resistance *R*_ct_, **d** the electrochemical porosity, **e** shielding-increase coefficient of FSA and FSA-based absorbers after immersion in 5.0 wt% NaCl solution, **f** anti-corrosion mechanism of the FSA@ZnO@Al_2_O_3_ gradient structure

Additionally, to balance the rate of anodic and cathodic polarization, the OCP curves were measured in 5.0 wt% NaCl solution for ~ 25–26 min, as shown in Fig. S10. It is found that the OCP values after 25 min for pure FSA, FSA@Al_2_O_3_, FSA@ZnO@Al_2_O_3_ are -0.01, 0.05, 0.03, and 0.12 V, respectively; a clear shift toward the direction of positive potential for FSA@ZnO@Al_2_O_3_ gradient structure. These results suggested that FSA-based absorbers are more inert than bare FSA in the NaCl solution, i.e., oxide shells form a very good anti-corrosion resistant barrier on FSA particles, especially ZnO/Al_2_O_3_ dual-oxide shell. The *I*_corr_ values for pure FSA, FSA@Al_2_O_3_, FSA@ZnO@Al_2_O_3_ are 8.196, 1.653, 0.8275, and 0.8351 μA cm^−2^, respectively, which demonstrate that the corrosion rate decreases clearly, displayed in Fig. 5a. Furthermore, the charge transfer resistance *R*_ct_ shows an increasing trend, indicating the enhanced protection for the dual-oxide shell, as shown in Fig. 5c. The corrosion rates (CR) are calculated by the followed equation $CR~\left( {\mu m/year} \right)~ = \frac{{M \times I_{{corr}} }}{{n\rho F}} \times 87600$ [55]. Where M is 55.85 g mol^−1^ of the formula weight, *n* is 2 of the chemical valency for Fe element, ρ is 7.86 g cm^−3^ of the density of Fe, *F* is 26.8 Ah of the Faraday constant. Figure 5b shows the calculated corrosion rates of these samples, it is observed that the CR value of FSA@ZnO@Al_2_O_3_ shows an obvious decline compared to pure FSA, revealing that its long-term corrosion protection. Generally, the porosity and the shielding-increase coefficient (β) are the significant parameters to evaluate the barrier effect. The porosity =${\text{R}}_{{{\text{ct}}}}^{{\text{0}}} {\text{/R}}_{{{\text{ct}}}}^{{\text{c}}} *{\text{100\% }}$ (${\text{R}}_{{{\text{ct}}}}^{{\text{0}}}$, cathodic charge transfer resistances of FSA, ${\text{R}}_{{{\text{ct}}}}^{{\text{c}}}$, that of FSA-based absorbers). The porosity values in Fig. 5d show a remarkably decline trend after different oxide deposition on FSA alloy, indicating the higher sealing efficiency, possibly attributed to a less defective and better nucleation growth of the oxide shell on the FSA surface [68]. Besides, the shielding-increase coefficient (β) is determined by the equation of β =|Z|_G0.01 Hz_/|Z|_O0.01 Hz_ (|Z|_G0.01 Hz_, |Z|_O0.01 Hz_ are the resistance values at 0.01 Hz of FSA-based absorbers and bare FSA, respectively). The bigger the β values, the better the barrier effect of ceramic oxide [69]. In Fig. 5e, it is found that the β value of FSA@ZnO@Al_2_O_3_ gradient structure is the highest among samples, suggesting that it possessed an excellent barrier effect to prevent FSA core from corrosion. Therefore, the high density and low porosity of ZnO/Al_2_O_3_ dual-oxide-shell could provide a good physical shielding effect to protect FSA alloy from corrosive media, such as Cl^−^, H_2_O, and O_2_, as shown in Fig. 5f.

## Conclusions

Here, we have designed a FSA@ZnO@Al_2_O_3_ gradient structure in a layer-by-layer fashion with atom-scale precision as an outstanding anti-corrosive microwave absorber through ALD technology. The as-synthesized gradient structure possesses a much lower RLmin and wider effective absorption bands than pristine FSA in low frequencies covering L and S bands. The RLmin of FSA@ZnO@Al_2_O_3_ gradient structure is as low as -50.6 dB, which is ~ 4 times lower than that of pristine FSA, and the effective absorption bands are 3 times wider than that of pristine FSA. The enhanced microwave absorption is attributed to dielectric loss from ZnO/Al_2_O_3_ dual-oxide-shells, interface relaxation, and Debye relaxation, magnetic loss from the inner FSA with DO_3_ superlattice structure, and the excellent impedance matching along with high attenuation characteristic. Moreover, the corrosion potential moves to 0.0191 V, the anodic current density is at least 1.0 orders of magnitude lower than that of pristine FSA and the corrosion rate decrease by about 9.0 times, confirming that the dual-oxide-shells are contributed to blocking the penetration of H_2_O, O_2_, and Cl^−^ into the FSA/ceramic layers interface. It is believed that the FSA@ZnO@Al_2_O_3_ gradient structure would be a promising material for the next-generation microwave absorbers with enhanced anti-corrosion properties.

## Supplementary Information

Below is the link to the electronic supplementary material.

Supplementary file1 (DOCX 4041 kb)
