# Supplementary material for: Atomic-Scale Layer-by-Layer Deposition of FeSiAl@ZnO@Al2O3 Hybrid with Threshold Anti-Corrosion and Ultra-High Microwave Absorption Properties in Low-Frequency Bands
Source: Nanomicro Lett. 2021 Jul 30;13:161. doi: 10.1007/s40820-021-00678-4 (PMC8324648; doi:10.1007/s40820-021-00678-4)
Supplement: Supplementary file 1 — Supplementary file1 (DOCX 4041 kb) [file 40820_2021_678_MOESM1_ESM.docx]

Supporting Information

Atomic-scale layer-by-layer deposition of FeSiAl@ZnO@Al_2_O_3_ hybrid with threshold anti-corrosion and ultra-high microwave absorption properties in low-frequency bands

Wei Tian^1, 2^, Jinyao Li^3^, Yifan Liu^3^, Rashad Ali^3^, Yang Guo^4^, Longjiang Deng^1,^ *, Nasir Mahmood^5,^ *, Xian Jian^1, 2, 3,^ *

^1^National Engineering Researching Centre of Electromagnetic Radiation Control Materials, Key Laboratory of Multi-Spectral Absorbing Materials and Structures of Ministry of Education, State Key Laboratory of Electronic Thin Films and Integrated Devices, School of Electronic Science and Engineering, University of Electronic Science and Technology of China, Chengdu, 611731, China.

^2^The Yangtze Delta Region Institute (Huzhou) & School of Electronic Science and Engineering, University of Electronic Science and Technology of China, Huzhou 313001, China.

^3^School of Materials and Energy, University of Electronic Science and Technology of China, Chengdu, 611731, China.

^4^School of Electrical and Information Engineering, Panzhihua University, Panzhihua, 617000, China.

^5^School of Engineering, RMIT University, Melbourne, 3001, Victoria, Australia.

Corresponding authors.

Prof. Jian Xian (jianxian@uestc.edu.cn)

Prof. Longjiang Deng (denglj@uestc.edu.cn)

Dr. Nasir Mahmood (nasir.mahmood@rmit.edu.au)

**
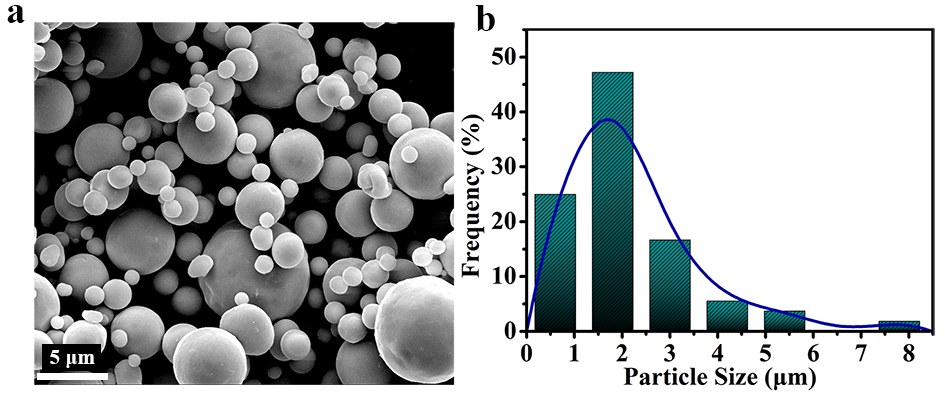
**

**Fig. S1** **a** SEM image of pure FSA, **b** Size distribution of FSA microspheres

**
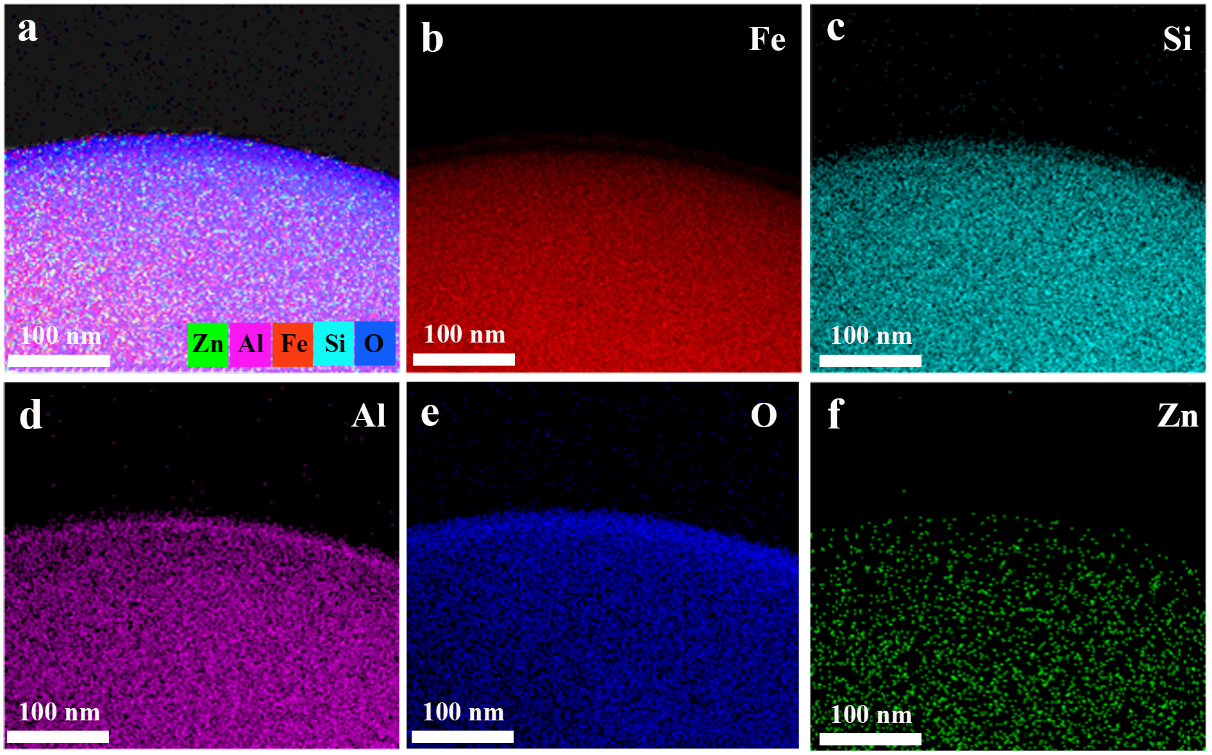
**

**Fig. S2** The elemental mapping images of FSA@ZnO@Al_2_O_3_ gradient structure: **a** overlap of all elements, **b** Fe, **c** Si, **d** Al, **e** O, and **f** Zn

**
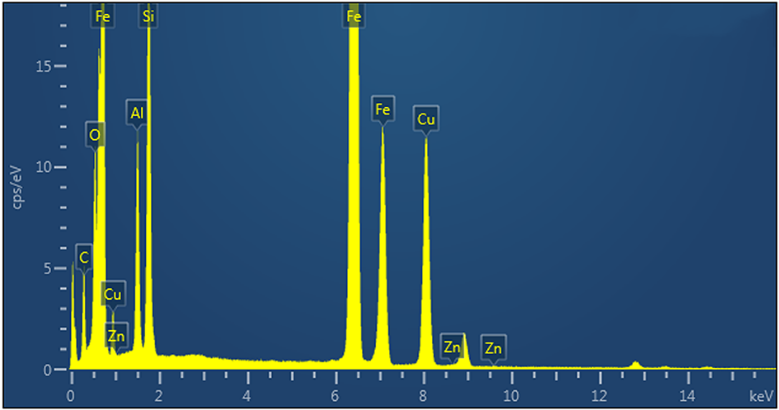
**

**Fig. S3** The EDS image of FSA@ZnO@Al_2_O_3_ gradient structure

**Table S1** The concentrations of different elements in FSA@ZnO@Al_2_O_3_ gradient structure

| Elements | The concentrations of different elements in FSA@ZnO@Al_2_O_3_ composites  (wt.%) |  |
| --- | --- | --- |
| Fe | 92.91 | |
| Si | 2.81 | |
| Al | 1.54 | |
| O | 2.44 | |
| Zn | 0.30 | |


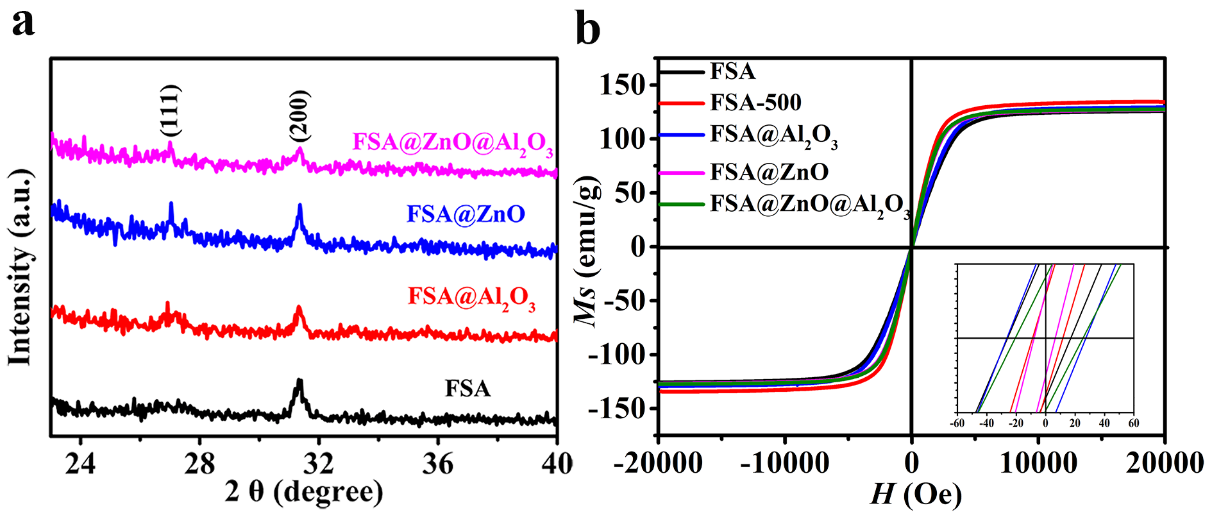


**Fig. S4 a** XRD patterns, **b** Hysteresis loops (*M-H* loops) of FSA, FSA-500, FSA@Al_2_O_3_, FSA@ZnO, and FSA@ZnO@Al_2_O_3_


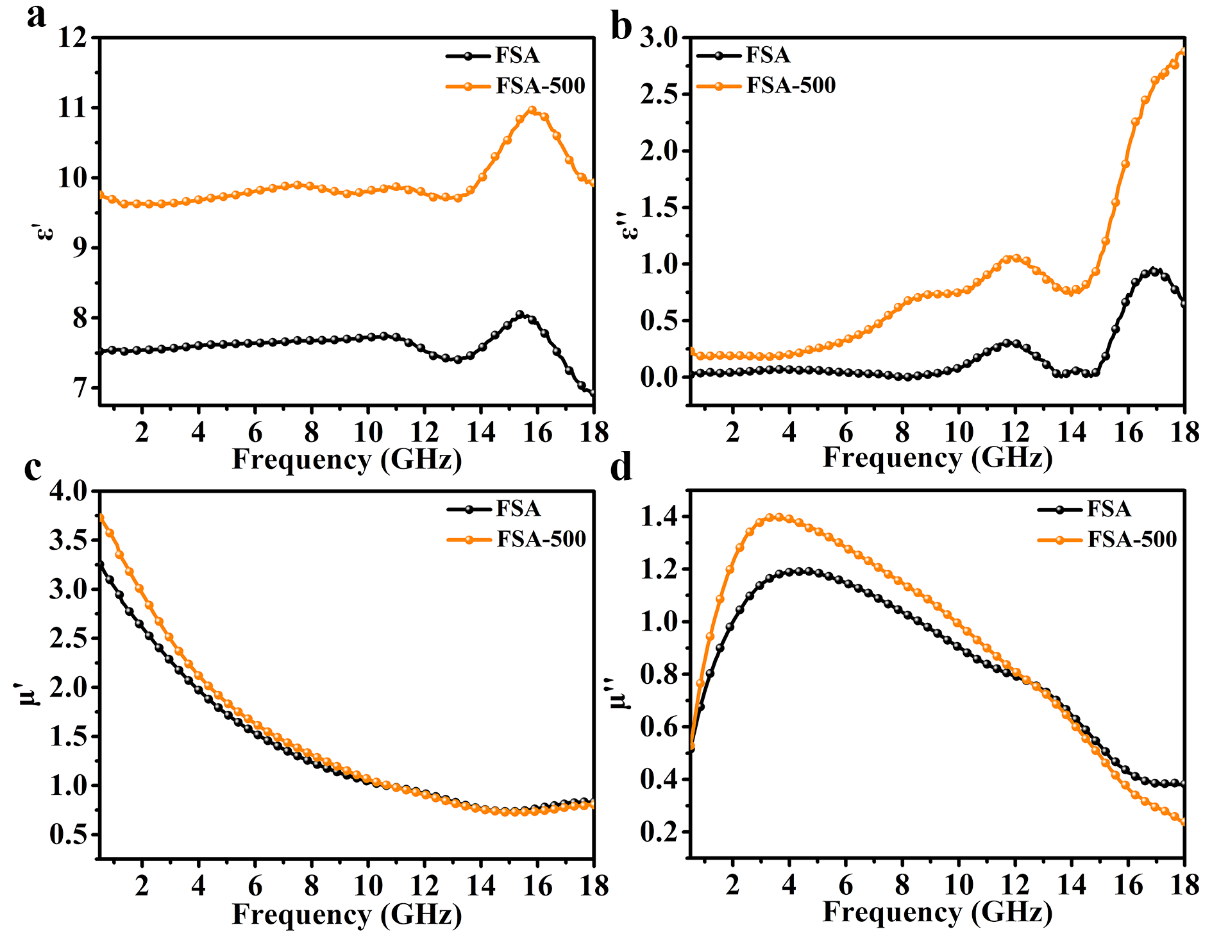


**Fig. S5** The frequency dependence of electromagnetic parameters of FeSiAl alloy and FeSiAl alloy annealed at 500 ℃ under an N_2_ atmosphere: **a** real parts (ε′), **b** imaginary parts (ε′′) of the complex permittivity, **c** real parts (μ′), **d** imaginary parts (μ′′) and magnetic loss tangents of the complex permeability


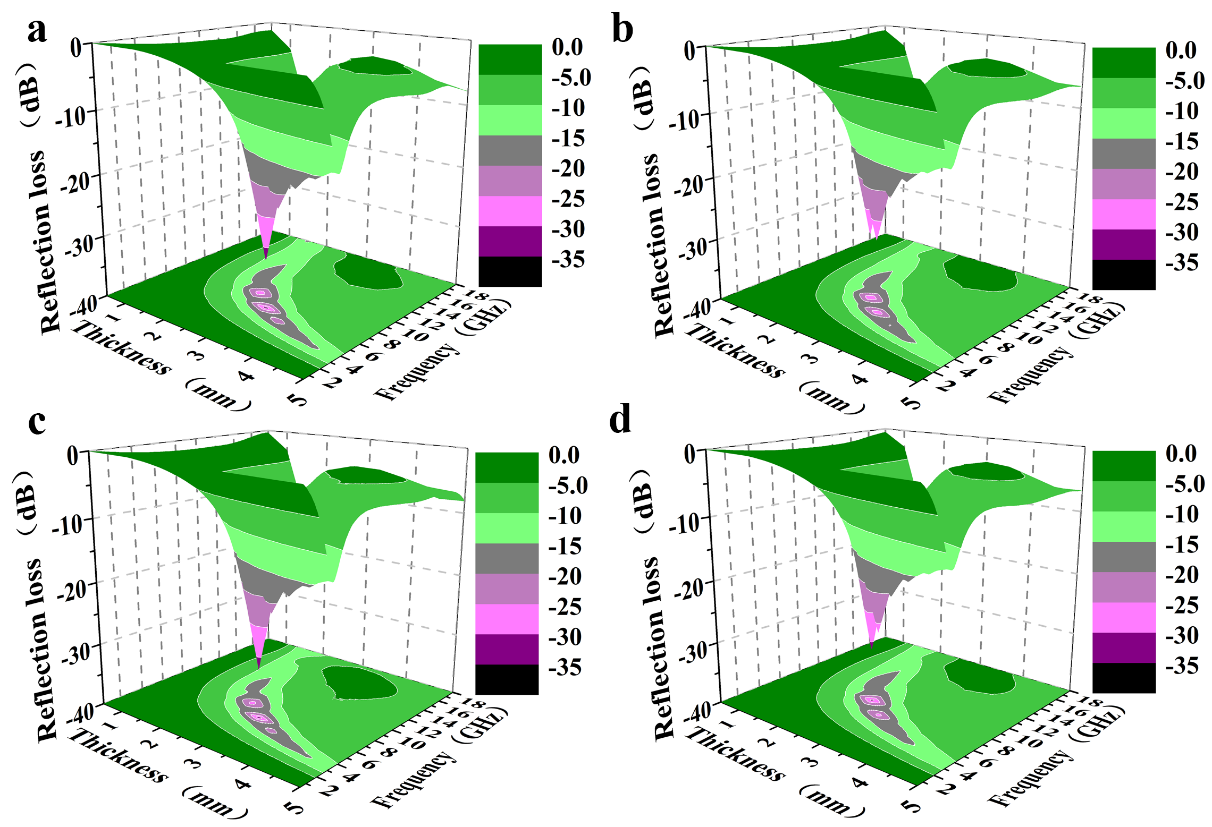


**Fig. S6.** 3D RL maps of as-prepared absorbers before heat treatment of d at 0.5–5.0 mm in 0.5–18 GHz: **a** FSA, **b** FSA@Al_2_O_3_, **c** FSA@ZnO, **d** FSA@ZnO@Al_2_O_3_

**Note S1**

A delta-function tool is proposed to investigate the degree of impedance matching between the absorbers and free space by the following equation [S1]:

$\left| \Delta\right|=\left| \sin h^{2}\left( Kfd \right)-M \right|$ (S1)

where K and M are calculated with the ε_r_ and μ_r_ in the following equations:

$\text{K=}\frac{\text{4π}\sqrt{\text{μ}^{\text{'}}\text{ε}^{\text{'}}}\sin\frac{\text{δ}_{\text{e}}\text{+}\text{δ}_{\text{m}}}{\text{2}}\text{ }}{\text{c}\cos\text{δ}_{\text{e}}\cos\text{δ}_{\text{m}}}$ (S2)

$\text{M=}\frac{\text{4}\text{μ}^{\text{'}}\text{cos}\text{ δ}_{\text{e}}\text{ ε}^{\text{'}}\text{cos }\text{δ}_{\text{m}}}{\left( \text{μ}^{\text{'}}\text{cos }\text{δ}_{\text{e}}\text{-}\text{ε}^{\text{'}}\text{cos }\text{δ}_{\text{m}} \right)^{\text{2}}\text{+}\left[ \tan\left( \frac{\text{δ}_{\text{m}}}{\text{2}}\text{-}\frac{\text{δ}_{\text{e}}}{\text{2}} \right) \right]^{\text{2}}\left( \text{μ}^{\text{'}}\text{cos }\text{δ}_{\text{e}}\text{+}\text{ε}^{\text{'}}\text{cos}\text{ δ}_{\text{m}} \right)^{\text{2}}}$ (S3)

The attenuation constant α is calculated by ε_r_ and μ_r_ according to the following [S2]:

$\text{α=}\frac{\sqrt{\text{2}}\text{π}\text{f}}{\text{c}}\text{×}\sqrt{\left( \text{μ''ε''}\text{-}\text{μ'ε'} \right)\text{+}\sqrt{{\text{(}\text{μ''ε''-μ'ε'}\text{)}}^{\text{2}}\text{+}{\text{(}\text{μ'ε''+μ''ε'}\text{)}}^{\text{2}}\text{ }}}\text{ }$ (S4)

According to Debye dipolar relaxation theory, the complex permittivity (*ε_r_*) can be explained through the following equation [S3]:

$\text{ε}_{\text{r}}\text{=}\text{ε}^{\text{'}}\text{+}\text{i}\text{ε}^{\text{''}}\text{=}\text{ε}_{\text{∞}}\text{+}\frac{\text{ε}_{\text{s}}\text{-}\text{ε}_{\text{∞}}}{\text{1+i}\text{ωτ}}\text{ }$ (S5)

Where ε_∞_, ε_s,_ and τ_0_ are the dielectric constant at infinite frequency, the relaxation time, and the static dielectric constant, respectively. From Equation. (2) and (3), they are expressed that

$\text{ ε}^{\text{'}}\text{=}\text{ε}_{\text{∞}}\text{+}\text{ }\frac{\text{ε}_{\text{s}}\text{-}\text{ε}_{\text{∞}}}{\text{1+}\text{w}^{\text{2}}\text{τ}^{\text{2}}}\text{ωτ}$ (S6)

$\text{ε}^{\text{''}}\text{= }\frac{\text{ε}_{\text{s}}\text{-}\text{ε}_{\text{∞}}}{\text{1+}\text{w}^{\text{2}}\text{τ}^{\text{2}}}\text{ωτ }$ (S7)

Furthermore, it can be finally deduced as below:

${\text{ }\left( \text{ ε}^{\text{'}}\text{-}\frac{\text{ε}_{\text{s}}\text{+}\text{ε}_{\text{∞}}}{\text{2}} \right)}^{\text{2}}\text{+}\left( \text{ ε}^{\text{''}} \right)^{\text{2}}\text{=}\left( \frac{\text{ε}_{\text{s}}\text{-}\text{ε}_{\text{∞}}}{\text{2}} \right)^{\text{2}}$ (S8)

Thus, the ε″ versus ε′ plot should contain many single semicircles, usually denoted as the Cole−Cole semicircle and each semicircle on behalf of a Debye dipolar relaxation.

Based on the Debye theory, ε″ can be expressed as in the follwing equation.

$\text{ε}^{\text{''}}\text{(ω)= }\text{ε}_{p}^{\text{''}}+\text{ε}_{c}^{\text{''}}=\frac{\text{ε}_{\text{s}}\text{-}\text{ε}_{\text{∞}}}{\text{1+}\text{ω}^{\text{2}}\text{τ}^{\text{2}}}\text{ωτ+}\frac{\delta}{\text{ε}_{\text{0}}\text{ω}}$ (S9)

where ω is the angular frequency and σ refers to electrical conductivity. Therefore, ε″ is divided into two parts: ε_p_″ represents polarization relaxation loss and ε_c_″ is the conductive loss. The conductive loss, polarization loss, conductivity, and relaxation time are fitted by the nonlinear square fitting method [S4]. As presented in Fig. S7, the polarization relaxation loss and the conductive loss ability are both enhanced after decorated by ceramic oxides, especially, FSA@ZnO@Al_2_O_3_ gradient structure.


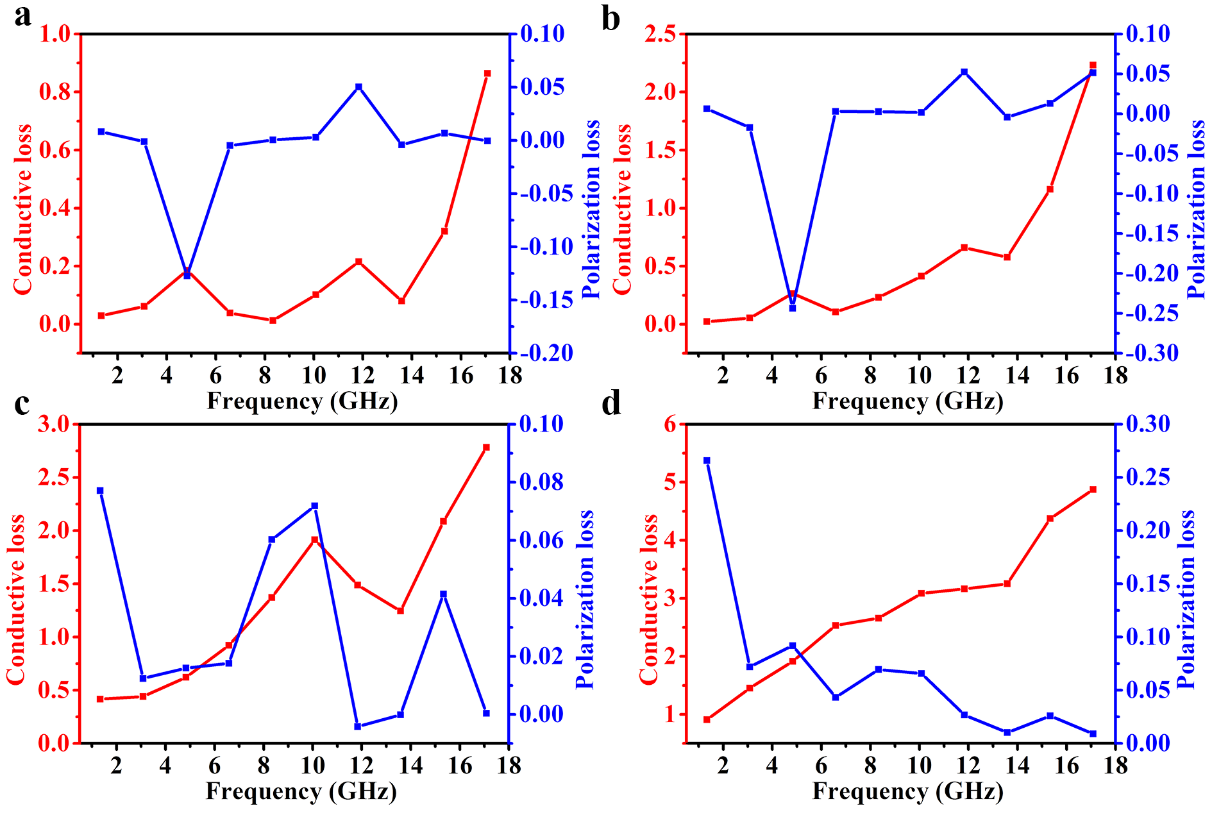


**Fig. S7** Plots of ε_c_″ and ε_p_″ vs frequency: **a** FeSiAl, **b** FSA@Al_2_O_3_, **c** FSA@ZnO, and **d** FSA@ZnO@Al_2_O_3_


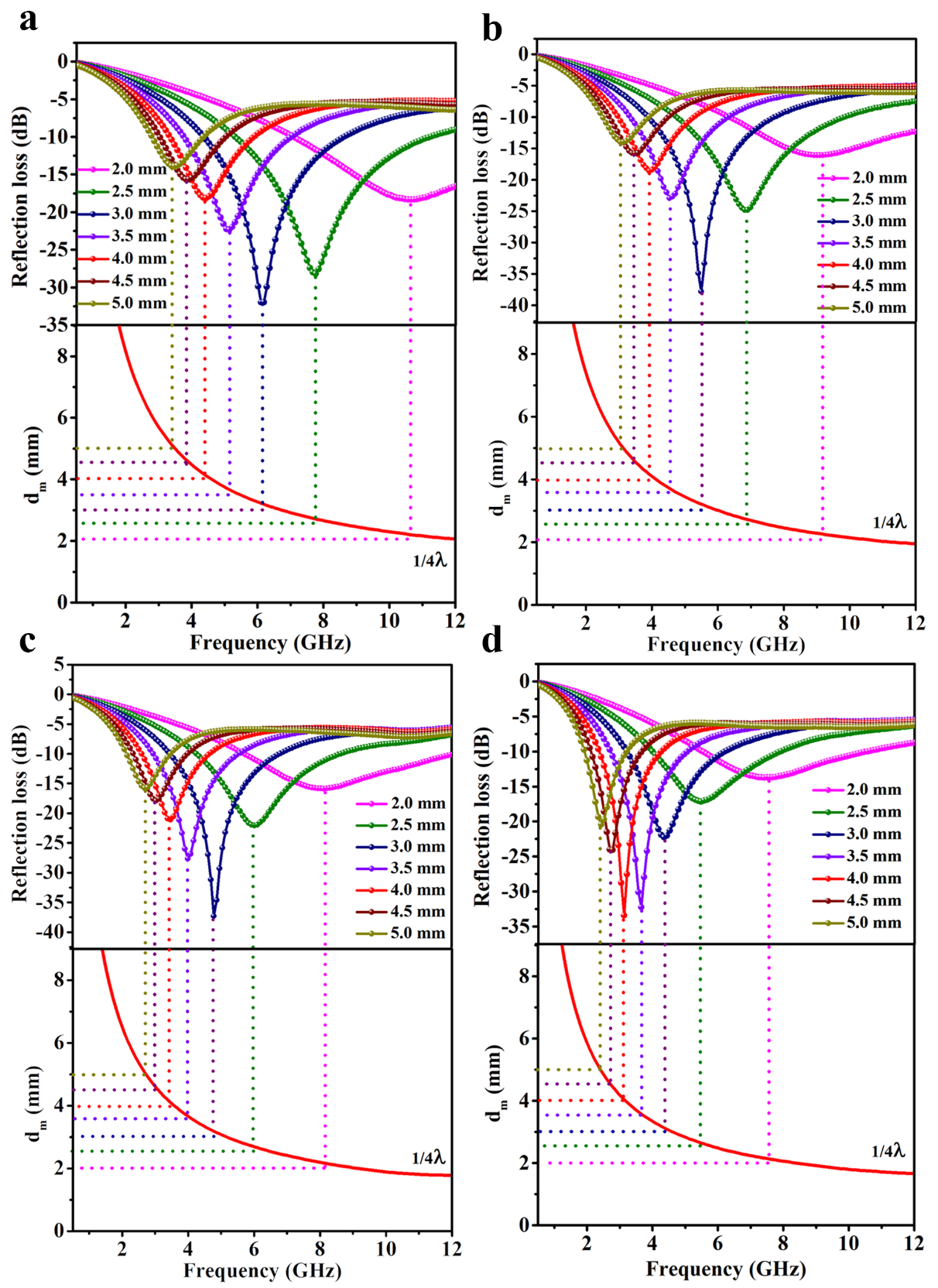


**Fig. S8.** RL curves and dependence of the absorber matching thickness (d_m_) versus matching frequency (f_m_) under wavelengths of λ/4 model for FSA-based samples: **a** FSA, **b** FSA@Al_2_O_3_, **c** FSA@ZnO, and **d** _­_FSA@ZnO@Al_2_O_3_ gradient structure

The equivalent circuit model is used to understand the effect of the electrolyte, oxide shell, and FSA electrochemical system, as shown in Fig. S9. In the circuits, R_s_ is the resistance of the NaCl solution, Q_A_ and Q_Z_ are the resistance of Al_2_O_3_ and ZnO shell, respectively. Q_A_ and Q_Z_ correspond to the capacitance of Al_2_O_3_ and ZnO shells, presenting a constant phase element capacitance. Also, Q_dl_ is the interface capacitance between the oxide shell and FSA core using a constant phase element, R_ct_ is the charge transfer resistance, and W is the Warburg impedance related to the diffusion of the electro-active particles in the system. The calculated circuit parameters are displayed in Table S2. An increase in R_ct_ values and a decrease in Q_dl_ values are observed in FSA-based absorbers, especially FSA@ZnO@Al_2_O_3_ gradient structure, indicating that high levels of corrosion resistance are provided by oxide shell, especially dual-oxide shell. Furthermore, the coating resistance and capacitance are contributed to protecting the FSA core from H_2_O, O_2,_ and Cl^-^ attacking.


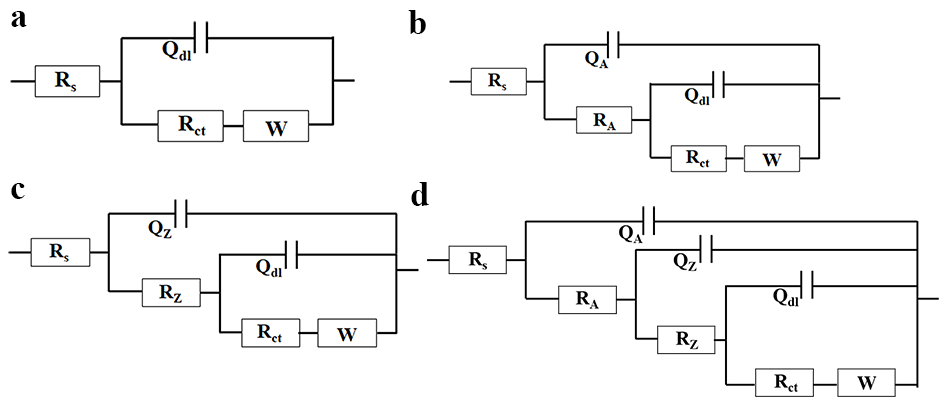


**Fig. S9** Equivalent circuit model used to fit the EIS data of a) bare FSA, **b** FSA@Al_2_O_3_, **c** FSA@ZnO, and **d** FSA@ZnO@Al_2_O_3_

**Table S2** Fitted Equivalent Circuit Model Parameters of FSA, FSA-based absorbers

| parameters |  | | FSA | |  | | FSA@Al_2_O_3_ | |  | | FSA@ZnO | |  | | | FSA@ZnO@Al_2_O_3_ | | |  |  |
| --- | --- | --- | --- | --- | --- | --- | --- | --- | --- | --- | --- | --- | --- | --- | --- | --- | --- | --- | --- | --- |
| R_s_ (Ω.cm^2^)  Q_dl_ (S^n^Ω^-1^)  n  R_ct_ (Ω.cm^2^)  Q_A_ (S^n^Ω^-1^)  n  R_A_ (Ω.cm^2^)  Q_Z_ (S^n^Ω^-1^)  n  R_Z_ (Ω.cm^2^)  W (Ω.cm^2^) | |  | | 11.54 | |  | | 10.9 | |  | | 9.6 | |  | | | 9.2 | | |  |
|  |  |  | | 1.9×10^-4^ | |  | | 1.5×10^-5^ | |  | | 8.3×10^-6^ | | |  | | | 9.1×10^-6^ | | |
|  |  |  | | 0.76 | |  | | 0.78 | |  | | 0.81 | | |  | | | 0.82 | | |
|  |  |  | | 42530 | |  | | 66370 | |  | | 80990 | | |  | | | 99740 | | |
|  |  |  | | --- | |  | | 1.5×10^-4^ | |  | | --- | | |  | | | 2.5×10^-5^ | | |
|  |  |  | | --- | |  | | 0.74 | |  | | --- | | |  | | | 0.91 | | |
|  |  |  | | --- | |  | | 761.7 | |  | | --- | | |  | | | 545.8 | | |
|  |  |  | | --- | |  | | --- | |  | | 6.9×10^-5^ | | |  | | | 3.7×10^-8^ | | |
|  |  |  | | --- | |  | | --- | |  | | 0.82 | | |  | | | 0.33 | | |
|  |  |  | | --- | |  | | --- | |  | | 911.0 | | |  | | | 5.88 | | |
|  |  |  | | 2.1×10^-3^ | |  | | 2.1×10^-4^ | |  | | 1.9×10^-4^ | | |  | | | 1.7×10^-4^ | | |


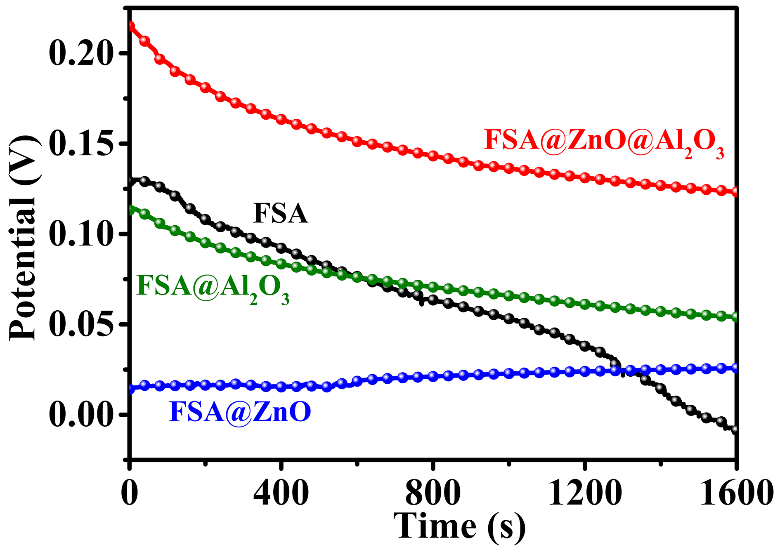


**Fig. S10** Open Circuit Potential *vs.* time curves of FSA and FSA-based absorbers after immersion in 5.0 wt.% NaCl solution

**Supplementary References**

[S1] L. Yan, C.Hong, B. Sun, G. Zhao, Y. Cheng et al., In situ growth of core–sheath heterostructural SiC nanowire arrays on carbon fibers and enhanced electromagnetic wave absorption performance. ACS Appl. Mater. Interfaces **9**, 6320-6331 (2017). https://doi.org/10.1021/acsami.6b15795

[S2] G. Z. Wang, X. G. Peng, L. Yu, G. P. Wan, S. W. Lin et al., Enhanced microwave absorption of ZnO coated with Ni nanoparticles produced by atomic layer deposition. J. Mater. Chem. A **3**, 2734-2740 (2015). https://doi.org/10.1039/C4TA06053A

[S3] X. Jian, W. Tian, J. Y. Li, L. J. Deng, Z. W. Zhou et al., High-temperature oxidation-resistant ZrN_0.4_B_0.6_/SiC nanohybrid for enhanced microwave absorption. ACS Appl. Mater. Interfaces **11**, 15869-15880 (2019). https://doi.org/10.1021/acsami.8b22448

[S4] H. Xu, X. Yin, M. Li, F. Ye, M. Han, et al., Mesoporous carbon hollow microspheres with red blood cell like morphology for efficient microwave absorption at elevated temperature. Carbon **132**, 343-351 (2018). https://doi.org/10.1016/j.carbon.2018.02.040
